# Bibliography

**Published:** 1912-08-15

**Authors:** 


### BIBLIOGRAPHY.

A MANUAL ON DENTAL HISTOLOGY: We have many excellent text books on general histology, but never until now have we had a book devoted entirely to dental histology in its practical or applied character. Dr. Frederick B. Noyes, for many years professor of histology in the Northwestern University Dental School, has put in book from for class work and general consultation the results of his research for the past ten years. As much of Dr. Noyes work has with the colaboration of Dr. G. V. Black been done on the structure of enamel and dentine with reference to cavity preparation, we find a most admirable statement of this phase of dental histology profusely illustrated with admirable cuts which supplement the text to good advantage. The illustrations all through the work are admirably done, and both the author and publishers deserve great credit for presenting this subject with so much detail. . The work also takes up the pulp, the peridental membrane and the alveolar process with equal care and graphic detail. The embryology and development of the jaws and teeth are clearly described and a good statement of bone development is also incorporated Directions for a laboratory course covering twenty-five periods is incorporated, giving directions for making, staining, mounting and examining specimens of tooth sections, etc, The work is timely and well done. We would suggest that for a complete text book more attention should be given to general histology, as this is important for the student, as a second text book on the subject will have to be bought by the student. The book is printed in the usual style and with the care of the well-known publishers, Lea & Febiger, Philadelphia.

